# Isolation and characterization of novel *RECK* tumor suppressor gene splice variants

**DOI:** 10.18632/oncotarget.5305

**Published:** 2015-09-28

**Authors:** Marina Trombetta-Lima, Sheila Maria Brochado Winnischofer, Marcos Angelo Almeida Demasi, Renato Astorino Filho, Ana Claudia Oliveira Carreira, Beiyang Wei, Thais de Assis Ribas, Michelle Silberspitz Konig, Christian Bowman-Colin, Sueli Mieko Oba-Shinjo, Suely Kazue Nagahashi Marie, William Stetler-Stevenson, Mari Cleide Sogayar

**Affiliations:** ^1^ Departamento de Bioquímica, Instituto de Química, Universidade de São Paulo, São Paulo 05508-000 SP, Brazil; ^2^ NUCEL-NETCEM-Faculdade de Medicina, Universidade de São Paulo, São Paulo, SP, 05360-120, Brazil; ^3^ Departamento de Bioquímica e Biologia Molecular, Universidade Federal do Paraná, Curitiba, PR, 81531-990, Brazil; ^4^ Dana Farber Cancer Institute, Harvard Medical School, Cambridge, MA, 02138, USA; ^5^ Radiation Oncology Branch, National Cancer Institute, National Institutes of Health, Bethesda, MD, 20892-4605, USA; ^6^ Departmento de Neurologia, Faculdade de Medicina, Universidade de São Paulo, São Paulo, SP, 01246-000, Brazil

**Keywords:** RECK, GBM, splicing, isoforms, MMP

## Abstract

Glioblastoma multiforme is the most common and lethal of the central nervous system glial-derived tumors. RECK suppresses tumor invasion by negatively regulating at least three members of the matrix metalloproteinase family: MMP-9, MMP-2, and MT1-MMP. A positive correlation has been observed between the abundance of *RECK* expression in tumor samples and a more favorable prognosis for patients with several types of tumors. In the present study, novel alternatively spliced variants of the *RECK* gene: *RECK-B* and *RECK-I* were isolated by RT-PCR and sequenced. The expression levels and profiles of these alternative *RECK* transcripts, as well as canonical *RECK* were determined in tissue samples of malignant astrocytomas of different grades and in a normal tissue RNA panel by qRT-PCR. Our results show that higher canonical *RECK* expression, accompanied by a higher canonical to alternative transcript expression ratio, positively correlates with higher overall survival rate after chemotherapeutic treatment of GBM patients. U87MG and T98G cells over-expressing the *RECK-B* alternative variant display higher anchorage-independent clonal growth and do not display modulation of, respectively, MMP-2 and MMP-9 expression. Our findings suggest that *RECK* transcript variants might have opposite roles in GBM biology and the ratio of their expression levels may be informative for the prognostic outcome of GBM patients.

## INTRODUCTION

Glioblastoma (GBM) is the most common, invasive and lethal tumor of the central nervous system. Gliomas are classified according to their morphological and histopathological characteristics into astrocytomas (approximately 67% of the cases), oligodendrogliomas and oligoastrocytomas [1–4]. Astrocytomas are classified by the World Health Organization using a scale of I–IV, with grade IV corresponding to GBM. One of the most striking features of GBMs is their enhanced potential to invade the normal surrounding brain tissue, rendering the tumor edges diffuse,during which the process of extracellular matrix (ECM) remodeling is essential [2, 5].

Matrix metalloproteinase (MMP) family members belong to the group of Zn^2+^-dependent endoproteases, which are key elements responsible for ECM turnover, being directly implicated in tumor invasiveness and metastasis. ECM degradation by MMPs not only facilitates tumor invasion, but also affects the behavior of both tumor and adjacent cells, leading to cancer progression. In general, the relative levels of MMPs increase with tumor progression [6]. A strong correlation between elevated expression levels of MMP-2, MMP-9, as well as membrane-associated MMPs, with higher grade astrocytomas has been described [3, 4, 6–8], suggesting that inhibitors of these MMPs may modulate astrocytoma progression.

To date, only one transcript, encoding a 110-kDa GPI membrane-anchored protein, has been described for the *REversion-inducing Cysteine-rich protein with Kazal motifs* (*RECK*) gene. The RECK canonical protein suppresses invasion, angiogenesis, and metastasis by negatively regulating at least three MMPs: MMP-9, MMP-2, and MMP-14 [9–12]. Other RECK targets have been identified, such as the extracellular metalloproteinases ADAM10 and CD13/aminopeptidase N [13, 14]. Recent studies also implicate RECK in stabilizing focal adhesions and anterior-posterior polarity [15]. Also, over-expression of *RECK* in several tumor-derived cell lines leads to suppression of the invasive and metastatic activity of these cells [12]. A positive correlation has been observed between the abundance of *RECK* expression in tumor samples and a more favorable prognosis for patients with several types of tumors, such as gastric, lung, pancreatic and colorectal cancer [16, 17].

It has previously been reported that downregulation of the *RECK* gene is critical for the invasive potential displayed by T98G GBM cells [18, 19], indicating the involvement of this gene in GBM biology. Nevertheless, the molecular pathway by which the *RECK* gene product exerts its actions is yet to be completely understood.

At least 60% of mammalian genes are subject to alternative splicing of pre-messenger RNA, adding complexity and flexibility to genomic expression, generating protein diversity and playing a key role in both physiological and pathological processes [20]. In the present study, two novel alternative transcripts of the *RECK* tumor suppressor gene, namely: *RECK-B* (1,548bp) and *RECK-I* (1,101bp), were identified and isolated by RT-PCR and their expression profiles were investigated using quantitative real time PCR (qRT-PCR) assays in both human astrocytomas of different malignant grades and in a normal tissue RNA panel. Our analysis shows that *RECK* transcript variants display different patterns and levels of RNA expression in samples from normal tissues, when compared to those of the canonical form. In GBM samples, higher expression of the *RECK* canonical, full length form, as well as a higher expression ratio of canonical to alternative transcripts expression were associated with better overall patient survival. In addition, over-expression of one of the characterized isoforms in the GBM cell line U87 MG, namely *RECK-B*, results in an enhanced rate of anchorage-independent cell growth. These findings suggest that RECK-B might have pro-oncogenic characteristics and, also, that the balance of the various transcripts may be prognostically informative for patients diagnosed with GBM.

## RESULTS

### *RECK* Tumor suppressor alternatively spliced variants

The human *RECK* gene spans an 87-kbp region on 9p13.3, and the canonical full-length transcript consists of 21 exons (NM_021111). Based on the analysis of Expressed Sequence Tags (ESTs) and non-reference sequences, available in the GenBank, we identified two novel putative splice variants of the *RECK* gene, which were named: *RECK-B* (1,548bp) and *RECK-I* (1,101bp). Prior to our study, only one Accession Number, NCBI's Genbank NM_021111 and Ensembl transcript ID ENST00000377966, was available for the *RECK* gene as a Reference Sequence, corresponding to the canonical form. But the sequence for transcript *B* had already been deposited as a non-Reference Sequence, Accession Number at the NCBI's Genbank CR593801.1. The *RECK-I* partial sequence is first described in the present study (GenBank ID 1520617).

**Figure 1:**
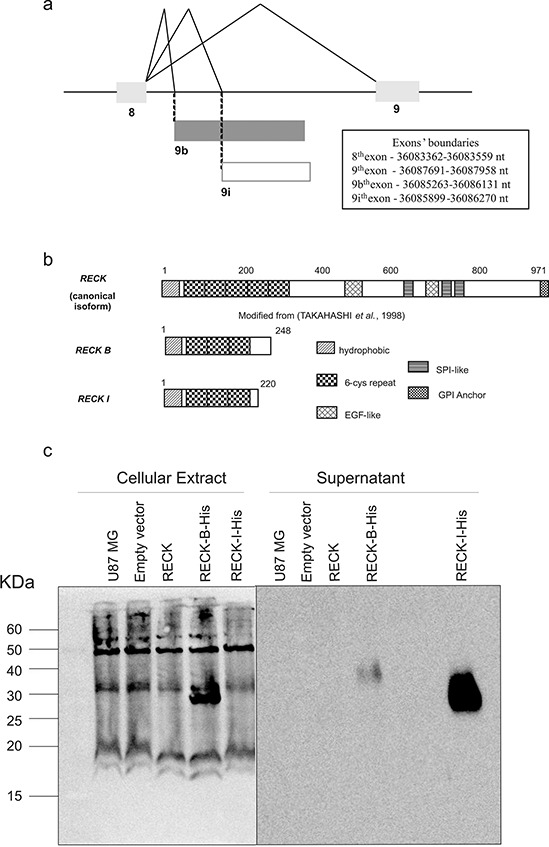
Description of RECK variants **a.** Representation of *RECK* gene alternative splicing mRNAs focusing on exons 8 and 9. The 8^th^ exon is shared by all three *RECK* variants, but the 9^th^ exon is unique to each variant, being generated through alternatively spliced acceptor sites; 9 represents the 9^th^ exon of the *RECK's* canonical form; 9b represents the 9^th^ exon of *RECK-B* variant; 9i represents the 9^th^ exon of the *RECK-I* variant; exons are represented by boxes and introns by lines; exons boundaries are specified based on Genbank assembly. **b.** Schematic representation of the predicted RECK protein isoforms. Numbers of amino acid residues are indicated. Analysis generated using PROSITE. **c.** Western Blot analysis of RECK-B and –I recombinant proteins fused with a c-terminal His-Tag in transfected U87 MG cells, detected in both the cellular extract (left panel) and in the cells supernatants (right panel).

Sets of primers were designed to amplify the sequences corresponding to the *RECK* alternative transcripts using total RNA from melanoma 1205 Lu cells. Both alternative variants consist of nine exons, sharing the first eight exons with the canonical transcript, whereas the ninth exon is final to each splice variant, being different from the corresponding exon in the canonical form and also different between the splice variants. This is due to the presence of alternative acceptors splicing sites at the 3′-intron/exon 9 boundary, giving rise to alternative exons. Diagrams of the corresponding *RECK* splice variants are shown in Figure 1a, together with the exons boundaries for each transcript variant.

The exon/intron boundary sequences of these transcripts show that the new splice sites involving the exons 8 and 9 junction of the novel transcripts are in accordance with the consensus sequences (AAG|GU or CAG|GU at the 5′-exon/intron boundary and CAG|GU at the 3′-intron/exon boundary) [21] with a few deviations (Table 1, Figure 1a). Interestingly, only the *RECK-I* alternative acceptor site was predicted by HSF (Human Splice Finder), with a MaxEnt matrix 3′-score of 8.47, while none of above mentioned alternative acceptor sites were predicted by ExonScan [22–25], suggesting that these *RECK* splice variants display weak alternative exon8/exon9 junction acceptor sites.

**Table 1:** Splice site boundaries of the *RECK* transcript variants

Variant	Donor exon (nt)	Acceptor exon (nt)	Intron size /Kb	EXON/intron/EXON (splicing site consensus: AG|guragu… (y)_10–15_nyag|G)
*RECK*	8 (198)	9 (318)	4.1	GAUA|**gu**aagu… uuauauucugaaaaugu**ag|G**UUUA
*RECK-B*	8 (198)	9 (818)	1.7	GAUA|**gu**aagu… gauaucuuuuaaaaauu**ag|G**GCCU
*RECK-I*	8 (198)	9 (371)	1.7	GAUA|**gu**aagu… cuuuauauauuuuccac**ag**|UGUUU

Donor and acceptor exons are listed with their sizes in nucleotides (nt) in brackets. Consensus nucleotide sequences at the 5′and 3′splice sites are indicated by bold red letters. Splice sites are indicates by vertical lines.

The nucleotide sequences of the two variants were then examined for their potential to encode valid open reading frames (ORFs) through NCBI's ORF Finder (Open Reading Frame Finder). Both transcripts were predicted to code proteins. Both predicted alternative isoforms share the ATG start codon with the canonical form, in which the open reading frame starts at exon 1, but each variant presents alternative stop codons within their ninth exon; therefore, each one displays a different stop codon. The *RECK* transcript variants encode smaller proteins than the canonical form, which has 971 amino acids. Thus, RECK-B and RECK-I are predicted to have 248 and 220 amino acid residues, respectively. Both proteins are predicted to share their first 213 amino acids with each other and with the canonical form. Also, both have distinct C-terminal sequences (Figure 1b). The predicted RECK isoforms sequences are provided as Supplementary material (Figure S1). Analysis of RECK-B and RECK-I amino acid sequences showed that the Epidermal Growth Factor-like and the Serine Protease-like domains, found in the canonical protein, are not present in any of the predicted protein isoforms.

The recombinant RECK variants (ORF Finder Predicted ORFs) fused with a C-terminal eight His-Tag were expressed in U87MG cells, displaying molecular weights which are in accordance with their predicted values (Figure 1c). Interestingly, RECK-B is detected both in the cellular extract (27 KDa), and conditioned media (40 KDa) while RECK-I is only detected in the cell culture media (37 KDa). The difference observed between RECK-B's molecular weight in the cellular extract and conditioned media might be explained by post-translational modifications.

### Expression profile of the *RECK* tumor suppressor gene splice variants in human tissues

To examine the expression levels of *RECK* variants in a variety of normal human tissues, different primers, which specifically amplify each isoform, were generated. Each pair of primers targeted a region that included the exon8/exon 9 boundary, so that each pair of primers would specifically recognize one *RECK* variant (sequences provided in Table S1). The relative mRNA expression levels of each *RECK* alternative transcript were then examined in several human tissues by qRT-PCR.

**Figure 2:**
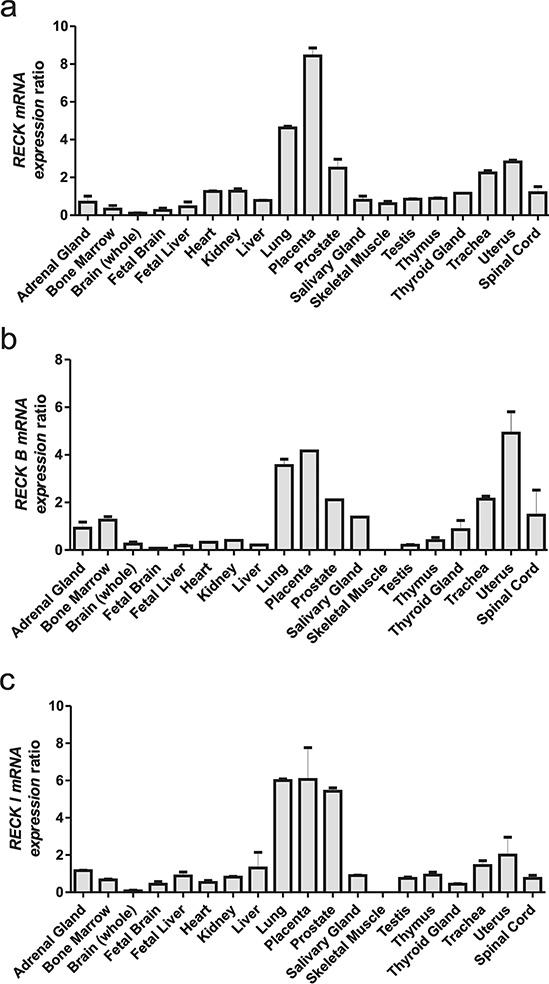
qRT-PCR analysis of the RECK gene mRNA isoforms across a Human Total RNA Master Panel II (Clontech) mRNA relative expression panel of each *RECK* alternative isoform: **a.** *RECK*, **b.** *RECK-B*, **c.** and *RECK-I*. *GAPDH* expression levels were used as an internal control for normalization. Data are represented as mean +/− SEM of two independent cDNA syntheses.

All *RECK* variants are expressed in normal human tissues, displaying a similar expression pattern amongst the three variants (Figure 2a–2c). However, there are a few significant differences, since *RECK* mRNA expression is higher in fetal brain when compared to adult brain and the same pattern is followed by *RECK-I*, but the RECK-B expression pattern is the opposite, being higher in adult brain. Also, it is interesting to note that the canonical variant is the only RECK transcript which has a detectable level in skeletal muscle. Taken together, these results indicate that *RECK* alternative transcripts may display divergent tissue-specific expression patterns in normal human tissues, when compared to the canonical transcript.

### Expression profiles of the *RECK* tumor suppressor gene splice variants, specific MMPs and TIMPs in astrocytomas of different grades of malignancy

We examined the mRNA expression profiles of *RECK* transcripts, as well as of MMPs reportedly inhibited by canonical RECK (*MMP-2, -9* and *-14)*, and of endogenous tissue inhibitors of MMPs (*TIMP-1, TIMP-2, TIMP*-*3* and *TIMP-4*) in astrocytomas of different grades of malignancy.

**Figure 3:**
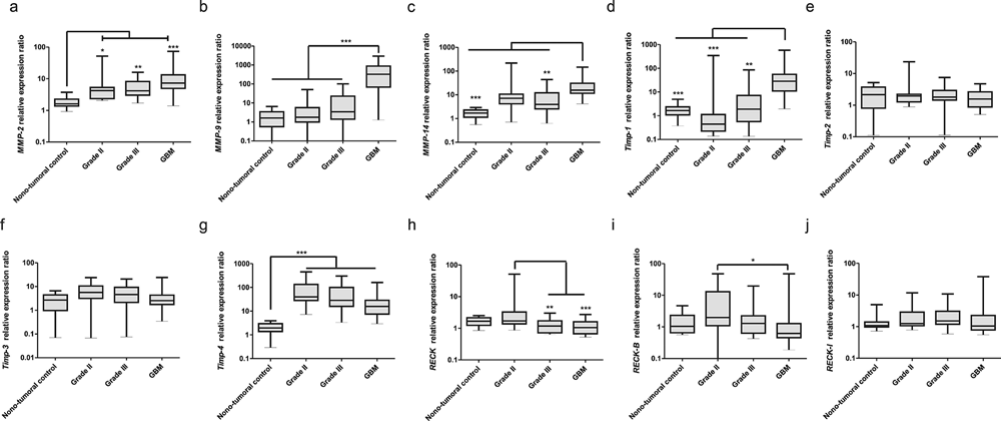
Expression profiles of MMPs, TIMPs and RECK variants in astrocytomas of different grades Brain samples taken from epileptic patients were taken as non-tumoral controls (*n* = 14), tumor samples were obtained from patients with astrocytomas grade II (*n* = 15), III (*n* = 15) and GBM (*n* = 30). qRT-PCR analysis of: **a.** *MMP-2*, **b.** *-9*, and **c.** *-14*; **d.** *TIMP-1*, **e.** *-2*, **f.** *-3* and **g.** *-4*; **h.** *RECK* canonical form, **i.** *RECK-B*, and **j.** *-I*. Expression levels of *GAPDH, HPRT* and *HMBS* were used as internal controls for normalization using the Genorm software. Non-parametric analysis of variance (Kruskal-Wallis test) followed by Dunn's test for *post hoc* comparison were used for statistical analysis. Data are represented as mean ± SEM. * represents *p* < 0.05; ** represents *p* < 0.01 and *** represents *p* < 0.001.

High expression of *MMP-2, -9* and *-14* was found in astrocytomas, when compared to the non-tumoral control group (Figures 3a–3c). Higher-grade astrocytomas display a sharp increase in *MMP-9* transcript levels (Figure 3b). Interestingly, it has been shown that *RECK* can directly modulate MMP-9 transcription levels [26]. *TIMP-2* is not differentially expressed among the different grade astrocytomas (Figure 3e). In contrast, *TIMP-1* expression displays a tendency to increase in association with higher tumor grades (Figure 3d), while *TIMP-3* and *-4* have a tendency to decrease (Figure 3f and 3g).

Canonical *RECK* expression decreases from grade II to higher-grade astrocytomas (Figure 3 h). In general, both alternative transcripts display more variable and/or dispersed expression pattern within a given histological grade, when compared to the canonical form. *RECK-B* showed a tendency to decrease from grade II astrocytomas to GBM, as does the canonical form (Figure 3i). *RECK-I* apparently is not modulated in this model (Figure 3j). Correlation analysis (Table 2) showed that the expression of all *RECK* transcripts correlates with each other and with *TIMP-2* expression. Canonical *RECK* expression also correlates with that of *TIMP-3* and *RECK-I* expression correlates with that of *MMP-2, MMP-14* and *TIMP-1*.

**Table 2:** Spearman non-parametric correlation test between relative mRNA expression levels

	RECK	RECK-B	RECK-I	MMP-2	MMP-9	MMP-14	TIMP-1	TIMP-2	TIMP-3	TIMP-4
*RECK*		**r = 0.4182;** ***p* < 0.0001**	**r = 0.4364;** ***p* < 0.0001**	r = 0.1680; ns	r = −0.0442; ns	r = 0.1838; ns	r = 0.0644; ns	**r = 0.5413;** ***p* < 0.0001**	**r = 0.3969;** ***p* < 0.0001**	R = 0.1135; ns
*RECK-B*	**r = 0.4182;** ***p* < 0.0001**		**r = 0.2782;** ***p* = 0.0083**	r = 0.0881; ns	r = 0.008483; ns	r = 0.0597; ns	r = 0.1285; ns	**r = 0.2657;** ***p* = 0.0119**	r = 0.0581; ns	r = 0.1349; ns
*RECK-I*	**r = 0.4364;** ***p* < 0.0001**	**r = 0.2782;** ***p* = 0.0083**		**r = 0.4303;** ***p* < 0.0001**	r = 0.1873; ns	**r = 0.4041;** ***p* < 0.0001**	**r = 0.3212;** ***p* = 0.0021**	**r = 0.2815;** ***p* = 0.0075**	r = 0.1156; ns	r = 0.1750; ns

ns stands for non-significant

### Implications of *RECK* splice variants expression in survival of glioblastoma patients

**Figure 4:**
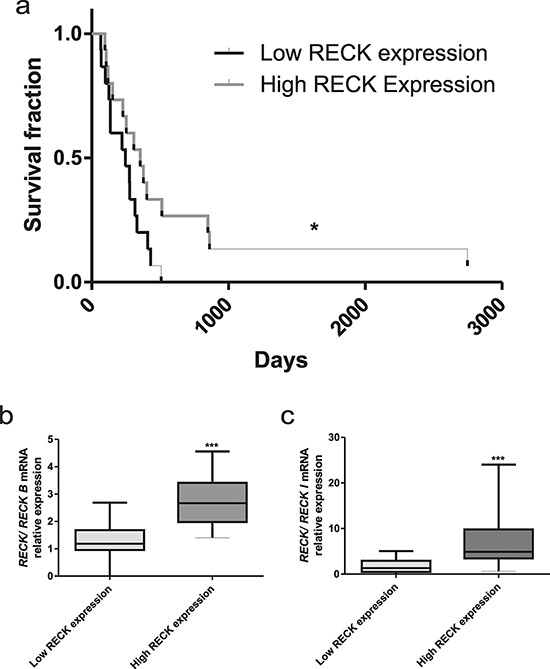
RECK expression and GBM patients survival rate **a.** Survival curves for GBM patients were divided into two groups, according to the levels of median canonical *RECK* mRNA expression, namely in: lower and higher *RECK* expression. **b.** Analysis of the ratios between canonical *RECK* and *RECK-B*, or **c.** canonical *RECK* and *RECK-I* expression. Mann-Whitney *t*-test was used for comparison between groups. Data are represented as mean ± SEM. * represents *p* < 0.05 and *** represents *p* < 0.001.

The RECK canonical form is a well-known favorable prognostic marker for a variety of tumors. In order to analyze the possible association between *RECK* transcripts expression and patient survival, the GBM (Grade IV) samples were divided into two groups, by the median value of the canonical *RECK* expression. We observed that the group of patients with higher canonical *RECK* expression displayed higher survival rates (Figure 4a). This group also displayed a higher expression ratio of canonical *RECK* transcript relative to each alternative variant (Figure 4b and 4c).

### Over-expression of the *RECK-B* alternative variant leads to increased U87 MG and T98G cells anchorage-independent growth

**Figure 5:**
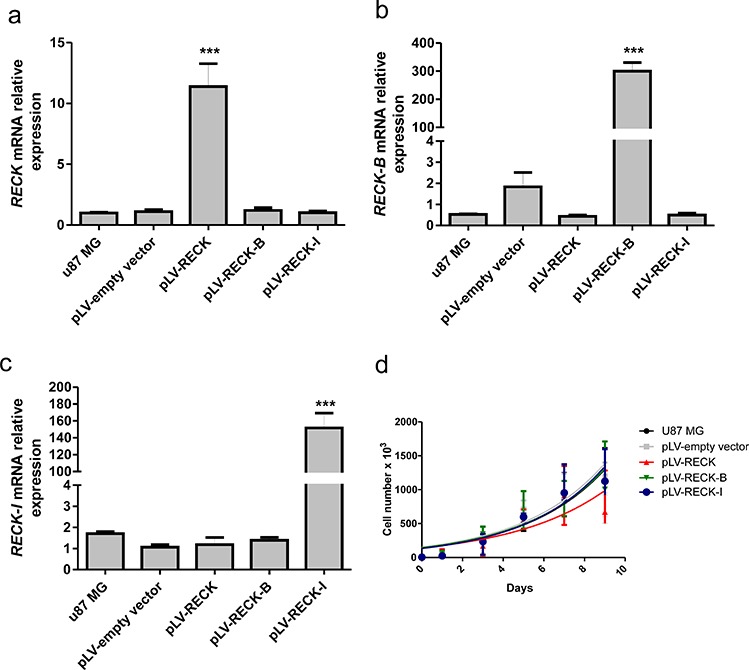
Validation of RECK variants over-expression and their effects in cellular growth rate U87 MG cells over-expressing *RECK, RECK-B* and *RECK-I*, as well as the empty vector control and parental cell line, were subjected to qRT-PCR expression analysis of: **a.** *RECK*, **b.** *RECK-B*, and **c.** *RECK-I* in order to validate their specific over-expression. **d.** Cells were subjected to growth curves, showing that *RECK* variants over-expression does not significantly alter U87 MG cells growth rate when cells were seeded in plastic substrate. ANOVA test followed by Tukey's test for *post hoc* comparison were used for statistical analysis. Data are represented as mean ± SEM. *** represents *p* < 0.001.

**Figure 6:**
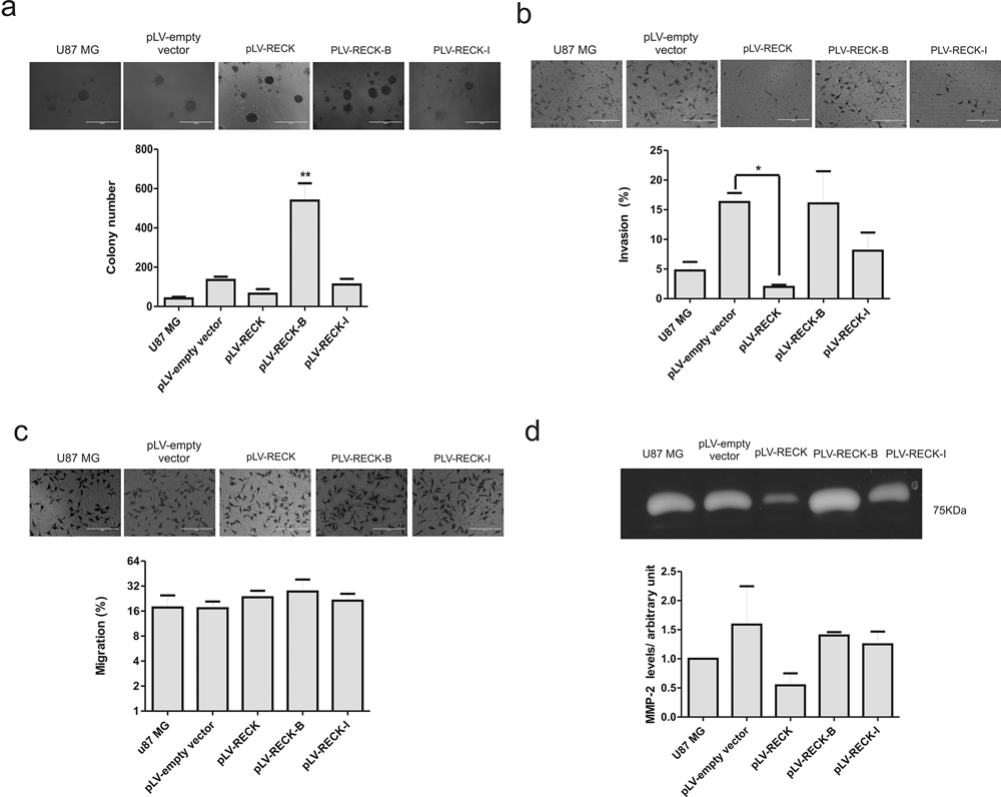
RECK variants functional analysis in U87 MG cells U87 MG cells over-expressing RECK, RECK-B and RECK-I, as well as the empty vector control, along with the parental cell line, were subjected to anchorage-independent clonal grow in semi-solid medium **a.** Invasion assay **b.** migration **c.** and gelatin zymography assay **d.** Results are represented as the mean with standard deviations from three independent experiments. ANOVA test followed by Tukey's test for *post hoc* comparison were used for statistical analysis. * represents *p* < 0.05 and ** represents *p* < 0.01.

In the present study, a higher survival rate was observed in patients with a higher abundance of canonical RECK expression when compared to its *B* and *I* variants. In order to better understand the role of the different RECK variants in GBM biology, each one of the RECK isoforms were overexpressed in U87 MG cells, and their over-expression was confirmed by qRT-PCR (Figure 5a–5c). The cell proliferation of U87 MG cells over-expressing each one of RECK isoforms in both monolayer and semi-solid medium were then assessed by growth curves (Figure 5d) and clonogenicity assays (Figure 6a). Although over-expression of none of the RECK variants significantly altered the growth rate of U87 MG cells in monolayer (Figure 5d), RECK-B over-expression led to a significant increase in anchorage-independent clonal growth of these cells (*p* < 0.001) (Figure 6a). To verify if RECK-B effect upon anchorage-independent clonal growth was not cell line-specific, we overexpressed canonical RECK and RECK-B in T98G cells and repeated the experiment (Supplementary Figure S2a). As observed in U87 MG cells, RECK-B over-expression in T98G cells led to a significant increase in anchorage-independent clonal growth of these cells (*p* < 0.05) when compared to cells transfected with the empty vector and over-expressing canonical RECK, suggesting that RECK-B might have a pro-oncogenic function.

### RECK alternatively spliced variants do not display MMP-2 inhibitory activity and do not alter U87 MG invasion capacity

Canonical RECK over-expression in different tumor cell lines, including the GBM T98G cells, is extensively described in the literature and shown to inhibit their invasive capacity [18, 19]. To further analyze the role played by *RECK* variants in GBM biology, taking into consideration the involvement of canonical RECK in MMP inhibition, the cells invasive and migration potential were then measured by *in vitro* transwell assays (Figure 6b and 6c). As expected, RECK over-expression led to a significant (*p* < 0.05) decrease in U87MG invasive potential, whereas RECK variants B and I over-expression did not significantly cells’ invasive capacity. For the purpose of verifying whether this unaltered invasive potential of the cells over-expressing RECK-B and –I reflected any alterations in secreted gelatinases levels, gelatin zymography assays were performed with the conditioned media collected from the U87MG derived cell lines. Indeed, conditioned media from cells over-expressing RECK-B and –I did not present decreased MMP-2 levels, as was observed with RECK over-expression, although without statistical significance (Figure 6d), indicating that RECK-B and I variants lack the ability to inhibit secreted MMP-2 protein levels. In T98G cells, neither canonical RECK nor RECK-B over-expression led to alteration of MMP-9 protein in their conditioned media (Supplementary Figure S2b).

## DISCUSSION

The involvement of MMPs in glioma progression and acquisition of aggressive behavior and the use of MMPs and MMPs inhibitors in glioma diagnosis [7–9] have been extensively described in the literature. Here, we focus on the isolation and characterization of *RECK* tumor and metastasis suppressor gene splice variants, their interaction with MMPs and the resulting impact in astrocytoma biology.

Alternative or aberrant splicing has an important role in the development of several pathologies, including cancer. Strong evidence indicates cancer-specific splice variants in 316 human genes involved in cell cycle control, signal transduction pathways, apoptosis, angiogenesis, invasion, motility and metastasis [27–36]. These studies showed mutations in *cis*-splicing regulatory elements and alterations in the cellular splicing regulatory machinery, leading to changes in the splicing pattern of several cancer-related genes, such as *CD44, BRCA1, OsABI5*, and *SECISBP2* [27–30, 36–38].

In the present study, we report on the identification of two novel *RECK* gene splice variants, namely: *RECK-B* (1,548bp), and *RECK-I* (1,101bp), and the characteristics of their expression profiles in normal human tissues and astrocytoma of different grades (II–IV). The *RECK* canonical form has 21 exons, however, both alternative variants consist of only nine exons each, being generated by alternative acceptor splicing sites at the 3′-intron 8/exon 9 boundaries. Therefore, these splice variants share the first eight exons with the canonical transcript, whereas the ninth exon is different from the ninth exon of the canonical form and also different between the two variants. Interestingly, the HSF and ExonScan splice site recognition tools failed to predict the *RECK-B* alternative acceptor site at the exon 8/exon 9 junction, while only HSF predicted *RECK-I* alternative acceptor site through MaxEnt matrices, which is the same algorithm used by ExonScan [22, 23]. The identified transcripts encode the following proteins: RECK-B (248 aa, 40 KDa) and RECK-I (220 aa, 30 KDa).

The *RECK* gene alternative transcripts are expressed in a variety of human tissues, displaying different mRNA expression profiles relative to the canonical variant, as well as between the different splice variants, demonstrating that although the three characterized variants apparently display weak alternative splice sites, these transcripts are ubiquitously expressed in different normal human tissues. It has previously been reported that the canonical *RECK* plays an important role during neurogenesis, specifically, during development of the murine cerebral cortical tissue regulated by Notch signaling [39]. Our results showed that the expression pattern of the canonical *RECK* transcript, which was higher in fetal brain relative to adult brain, was opposite from that observed for *RECK-B*, suggesting that the balance of *RECK* transcripts and their differential modulation might play a role during embryogenesis of this tissue.

Classically, it is widely recognized that canonical RECK is involved in suppression of tumor invasion, angiogenesis and metastasis, in part, by negatively regulating at least three MMPs, namely: MMP-9, MMP-2 and MT1-MMP [9–12], as well as the extracellular metalloproteinases ADAM10 and CD13/aminopeptidase N [13, 14]. The SPI-like domains present in the canonical form confer to the RECK protein the ability to inhibit the MMP-9 activity [23]. Both identified RECK variants lack these SPI-like domains. Also, gelatin zymography assays of conditioned media from U87MG-derived cell lines over-expressing the canonical RECK display lower secreted MMP-2 amounts, but such a decrease was not observed in conditioned media when either RECK-B or RECK-I were overexpressed. In addition, gelatin zymography assays of conditioned media from T98G-derived cell lines over-expressing the canonical RECK or RECK-B did not display any changes in MMP-9 levels. Moreover, U87MG cells over-expressing RECK presented diminished cell invasion while *RECK-B* over-expressing cells demonstrated no impact in cell invasion whereas these cells displayed higher anchorage-independent clonal growth. Additionally, the canonical *RECK* expression levels positively correlated with *TIMP-3* expression, as we have previously reported in a melanoma progression model [40], and *RECK, RECK-B* and *RECK-I* expressions levels correlated positively with *TIMP-2*. The canonical RECK expression [41, 46] has been reported to be induced by TIMP-2 [21, 43], and based on the present observation of correlated expressions levels of both variants with *TIMP-2*, we speculate that these variants are also modulated by *TIMP-2*. Of note, no correlation was found between *RECK* variants expression and *MMP-9* expression, which is the only MMP transcriptionally regulated by the canonical RECK [26, 44]. Interestingly, both transcripts are regulated by the same microRNA, MiR-21 [45].

The invasive tumor behavior, which implies ECM remodeling [3], is a pathological hallmark of malignant astrocytomas, rendering the tumor edges diffuse and establishing countless micrometastasis, a feature responsible for difficulties of total tumor resection as well as post-surgical treatment, and that contributes to its frequent recurrence. Analysis of *RECK* alternative transcripts expression in astrocytomas of different malignant grades (II, III and IV/GBM) by qRT-PCR, revealed that, although *RECK-I* apparently was not modulated during astrocytoma progression, *RECK-B* displayed lower expression levels in GBM, when compared to grade II astrocytomas, a similar pattern to that of the canonical transcript, but with a more variable expression distribution within each tumor grade.

Upon alternative splicing processing, several genes which are relevant for cancer biology, may yield either oncoproteins or tumor suppressor proteins, therefore, the balance between the different isoforms does influence the outcome of certain molecular pathways and may be determinant of a pathological outcome. Classical examples are the Bcl-X and the VEGF genes. bcl-X codes for a pro- (Bcl-X_S_) and an anti- (Bcl-X_L_) apoptotic form, while VEGF codes for both pro- (VEGF-XXX) and anti-(VEGF-XXXb) angiogenic isoforms [46–50] Interestingly, when the ratio of canonical RECK expression/RECK-B was analyzed, GBM patients presenting higher expression of RECK according to this ratio presented longer overall survival, suggesting that RECK isoforms balance influence over GBM biology and disease outcome should be explored. The present result corroborates previous observation of positive correlation between the abundance of the canonical RECK expression and better prognosis of patients with gastric, lung, pancreatic and colorectal cancers [16, 17]. Taken together, our results suggest that the RECK-B transcript variant might present an opposite functional role, when compared to the canonical RECK, leading to increased anchorage-independent growth and, thus, stimulating malignancy traits, therefore, the balance of their expression levels may be informative relative to the influence of the *RECK* gene in GBM and, consequently, in patients prognosis.

## MATERIALS AND METHODS

### Cell culture, tissue samples collection and normal human RNA panel

1205 Lu cells were maintained in melanoma medium, consisting of four parts of MCDB153 (Sigma, St. Louis, MO) and one part of L–15 (Life Technologies, Carlsbad, CA), supplemented with 2 mM CaCl_2_, 5 μg/ml insulin (Sigma) and 2% fetal bovine serum (Invitrogen), 1% streptomycin/ampicillin and maintained at 37°C, 2% CO_2_ under controlled humidity. 293T (ATCC^®^ CRL–11268™), T98G (ATCC^®^ CRL–1690™), and U87 MG (ATCC^®^ HTB–14™) cells were maintained in Dulbecco's Modified Eagle's Medium (DMEM) supplemented with 10% fetal calf serum (FCS) at 37°C, 2% CO_2_ under controlled humidity. Both U87 MG and T98G are well-established models for the study of GBM biology and molecular pathways [51, 52]. U87 MG is a cell line established from a GBM sample from a 44 years old Caucasian male, highly invasive and able to produce tumors in nude mice [53, 54]. On the other hand, T98G is a cell line established from a GBM sample from a 61 years old Caucasian male, the cells undergo G1 phase cell cycle arrest under stationary conditions and are unable to produce tumors in nude mice, but present anchorage-independent growth [55]. Human Normal Total RNA Master Panel II (Lot. No. 7090015) was purchased from Clontech (Palo Alto, CA).

### Tissue samples from human subjects

Brain tissue samples from temporal lobotomy of epileptic patients and resected astrocytoma specimens were macrodissected and immediately snap-frozen in liquid nitrogen, as previously described [56]. The tumor specimens were categorized according to the WHO classification [1]. Written informed consent was obtained from all patients according to the ethical guidelines approved by the Ethics Committee of the School of Medicine, University of São Paulo (0600/10), and The Ethical Commission for Research Projects Analysis from the Clinical Board of The Clinical Hospital and School of Medicine, University of São Paulo (CAPPesq Approval No.: 830/01, CONEP/MS Approval No: 373/02).

### RNA isolation, cDNA synthesis and reverse transcriptase PCR

Total RNA was isolated using the RNeasy Mini Kit (Qiagen, Hilden, Germany). RNA quality control was evaluated through the 280/260 nm and 230/260 nm absorbance ratios and by observing the intensity ratio of the 18S and 28S rRNA bands under denaturing agarose gel electrophoresis. cDNA was obtained using Super Script III Reverse Transcriptase according to the manufacturer's instructions (Life Technologies).

### Rapid amplification of cDNA ends (RACE)

5′-RACE and 3′-RACE were carried out based on the 5′and 3′ RACE System for Rapid Amplification of cDNA Ends Kits (Life Technologies) according to the manufacturer's instructions. For the 5′-RACE, the first-strand cDNA was generated by reverse transcription of poly(A)^+^ RNA from 1205 Lu cells using the 5′-cDNA synthesis (CDS) primer, along with specific primers for the respective sequences (Table S1). For the 3′-RACE, the first-strand cDNA was generated using the 3′-CDS primer along with specific primers for the sequences. Sets of primers were designed using Primer3 (http://biotools.umassmed.edu/bioapps/primer3_www.cgi) and validated through BLAST and BLAT; sequences are shown in Table S1. The PCR products were purified, sub-cloned into a pGEM-T Easy plasmid (Promega, Madison, WI), and sequenced using the BigDye Terminator Cycle Sequence Ready Reaction Kit (Applied Biosystems, Foster City, CA).

### PCR amplification, cDNA sequencing and cloning

PCR amplification was carried out using the Phusion Hot Start High Fidelity DNA Polymerase (Finnzymes, Espoo, Finland), according to the manufacturer's recommendations. The sets of primers were designed using Primer3 and validated through BLAST and BLAT (Table S1). The PCR products were purified, sub-cloned into a pGEM-T Easy plasmid (Promega) and sequenced using the BigDye Terminator Cycle Sequence Ready Reaction Kit (Applied Biosystems). The *RECK* proteins coding sequences were amplified and cloned into the p156RRLsinPPCCMVIns3IRESPRC vector (a generous gift from Dr. Inder Verma, Salk Institute for Biological Studies, La Jolla, CA, USA). The coding sequences to each of the RECK isoforms were amplified using a primer containing the sequence to a C-terminal eight Histidine-Tag (His-Tag). These amplicons were then cloned into the pcDNA3.3-TOPO vector (Life Technologies), and, subsequently, the integrity of these constructs was assessed through DNA sequence analysis.

### Generation of a cell line enriched for EGFP expression

The p156RRLsinPPCCMVIns3IRESPRC *RECK* variants generated constructs were transduced into U87 MG cells using a third generation lentiviral system (Dr Inder Verma, Salk Institute for Biological Studies) according to Tiscornia and collaborators [57]. The positively transduced population, which expresses the EGFP reporter gene, was enriched (>96% of total population) by cell sorting, using a FACS Aria I/II cytometer (BD Biosciences).

### Western blotting

The pcDNA3.3-TOPO vectors, containing the coding sequences for the *RECK* gene variants, plus a C-terminal His-Tag, were transfected into 293T cells using the Lipofectamine 2000 reagent (Life Technologies), according to the manufacturers instructions, and, after 4 days of culturing, the cell culture medium was collected and the cellular extracts obtained by lysing the cells with RIPA buffer (150 mM NaCl, 1% NP-40, 0.5% SDS, 50 mM Tris pH8, 2 mM EDTA). 100 μL of these conditioned media were resolved on a 12.5% SDS-Page gel and transferred to a nitrocellulose membrane. The membranes were blocked for one hour in PBS-0.1% Tween 20 and 5% milk, followed by incubation for 1 h at room temperature with the Anti-His-Tag antibody (GE Helathcare, Buckinghamshire, UK) diluted 1:1,000 in PBS-0.1% Tween 20 (PBST) and 5% milk. After three washes of 10 min each with PBST, the membranes were incubated with the anti-mouse antibody (Qiagen) diluted 1:2,000 in PBST and 5% milk for one hour. Signal was detected the using Immobilon Western Chemiluminescent HPR substrate (Merck Millipore, Darmstadt, Germany).

### qRT-PCR

Non-tumoral brain control samples (*n* = 14) and samples of astrocytomas grade II (*n* = 15), astrocytomas grade III (*n* = 15), and GBM (*n* = 30) were analyzed. A panel of normal human tissue RNA was also analyzed. cDNA was amplified, in duplicate, using the Sybr Green dye (Applied Biosystems) in a GeneAmp 7300 Sequence Detection System (Applied Biosystems), according to the manufacturer's instructions. A dissociation cycle was performed after each run to check for non-specific amplification or contamination. A normalization value was generated through the geNorm program, using GAPDH, HPRT, and HMBS as housekeeping genes. Relative expression levels were estimated using a previously described method [58]. Sets of primers, specific for each RECK variant, TIMPs, and MMPs, were designed using Primer Express Software v.3 (Applied Biosystems), and validated through BLAST and BLAT. Sequences of all primers employed are presented in Table S1.

### Growth curve assays

Growth rates were established by seeding 5.10^4^ cells/well on a 6-well plate, the cells were allowed to grow for different periods of time and then collected and counted every 48 h. The cell number in each time point was plotted and the growth rate was determined as the angular coefficient of the fitted curve.

### Anchorage-independent clonal growth in semi-solid media

The wells of a 24 well-plate were coated with 500 μl 0.6% agarose (Fisher Scientific, Leicestershire, UK) solution in 10% FCS-DMEM. After agarose polymerization 1.10^4^ cells/well were then seeded on top of the 0.6% agarose and allowed to stand for about 10 min before the addition of 500 μl of melted 0.3% agarose in 10% FCS-DMEM. Finally, 500 μl of 10% FCS-DMEM were added. This liquid medium was renewed every two days and cells were allowed to grow for about 21 days forming large colonies, which were then quantified under a stereomicroscope.

### Gelatin zymography

MMP-2 activity present in the 48 h conditioned media of U87 MG cells over-expressing each one of the *RECK* variants was assessed by gelatin zymography. The conditioned media of the different cell lines were resolved in 10% SDS-polyacrylamide gels co-polymerized with 0.1% Porcine Gelatin A (Sigma, St. Louis, USA). After electrophoresis, the gels were washed for 15 min at room temperature with 2.5% Triton X-100, followed by incubation for 72 h at 37°C in a buffer containing 50 mM Tris-Cl (pH 8.5), and 10 mM CaCl_2_ and 1 μM ZnCl_2_ [42, 55]. The gels were stained with Coomassie blue R-250 (Sigma), 40% methanol (Merck), 10% acetic acid (Merck) and destained with 40% methanol, 10% acetic acid in water. Quantitative densitometry of the images of the negatively stained bands was performed using the ImageJ software (NIH, Bethesda, MD) and the values were normalized relative to the number of cells at the time of the collection.

### Migration and invasion *in vitro* assays

The migration and invasive potential of the U87 MG-derived cells over-expressing each one of the *RECK* variants were investigated by seeding 1.10^4^ cells on top of an uncoated or Matrigel-coated 8 μm pore transwells, respectively (BD Bioscience, Franklin Lakes, NJ). Cells were maintained in serum-free medium. As a chemo-attractive, the bottom chamber medium (DMEM) was supplemented with 10% FCS and cells were allowed to migrate or invade for 24 h. Cells remaining at the top chamber were removed and those present at the bottom of the filter were fixed with 3.7% formaldehyde (Sigma) for 20 min, permeabilized with methanol (Sigma) for 10 min and stained with Coomassie blue 250-R 0.125% in methanol: acetic acid: H_2_O (45:10:45, v/v/v) for 2 min. The number of migrating or invading cells per high power field (hpf) were counted using a microscope.

### Statistical analysis

Comparisons of the relative mRNA expression between different astrocytoma grade samples were carried out by a non-parametric analysis of variance (Kruskal-Wallis test) with Dunn test for post hoc comparison. The Spearman test was used for gene expression correlation analysis. The Mantel-Cox test was used for survival analysis. For comparison between subgroups of GBM samples, the Mann-Whitney's *t*-test was used. Comparisons of different groups, obtained in the clonal growth in semisolid media, migration, invasion and gelatin zymography, were carried out by the one way ANOVA variance analysis and the post hoc Tukey-Kramer test. All statistical analyses were carried out using the SPSS and Graph Pad Prism 4 programs. The results are presented as the mean with error bars standing for the standard error of the mean. A *p*-value of less than 0.05 was considered as statistically significant.

## SUPPLEMENTARY MATERIALS FIGURES AND TABLE


