# Supplementary material for: Isolation and characterization of novel RECK tumor suppressor gene splice variants
Source: Oncotarget. 2015 Sep 28;6(32):33120–33. doi: 10.18632/oncotarget.5305 (PMC4741753; doi:10.18632/oncotarget.5305)
Supplement: Supplementary file 1 [file oncotarget-06-33120-s001.pdf]

SUPPLEMENTARY FIGURES AND TABLE

RECK Isoform B ORF Finder Prediction (248aa):

M A T V R A S L R G A L L L L L A V A G V A E V A G G L A P G S A G A
L C C N H S K D N Q M C R D V C E Q I F S S K S E S R L K H L L Q R A
P D Y C P E T M V E I W N C M N S S L P G V F K K S D G W V G L G C
C E L A I A L E C R Q A C K Q A S S K N D I S K V C R K E Y E N A L F
S C I S R N E M G S V C C S Y A G H H T N C R E Y C Q A I F R T D S S
P G P S Q I K A V E N Y C A S I S P Q L I H C V N N Y T Q S Y P M R N
P T D **R** P P D E A A P E M A L Q S L R F V H P G I H F L H L E V T R F
I K T D

RECK Isoform I ORF Finder Prediction (220aa):

M A T V R A S L R G A L L L L L A V A G V A E V A G G L A P G S A G A
L C C N H S K D N Q M C R D V C E Q I F S S K S E S R L K H L L Q R A
P D Y C P E T M V E I W N C M N S S L P G V F K K S D G W V G L G C
C E L A I A L E C R Q A C K Q A S S K N D I S K V C R K E Y E N A L F S
C I S R N E M G S V C C S Y A G H H T N C R E Y C Q A I F R T D S S P
G P S Q I K A V E N Y C A S I S P Q L I H C V N N Y T Q S Y P M R N P
T D **S** R S V L S D I

Supplementary Figure S1: Predicted protein sequences encoded by the RECK alternative isoforms. *RECK B* and *RECK I*, generated by the ORF Finder (http://www.ncbi.nlm.nih.gov/projects/gorf/) program. The respective aminoacids at which the sequences start to differ are highlighted.

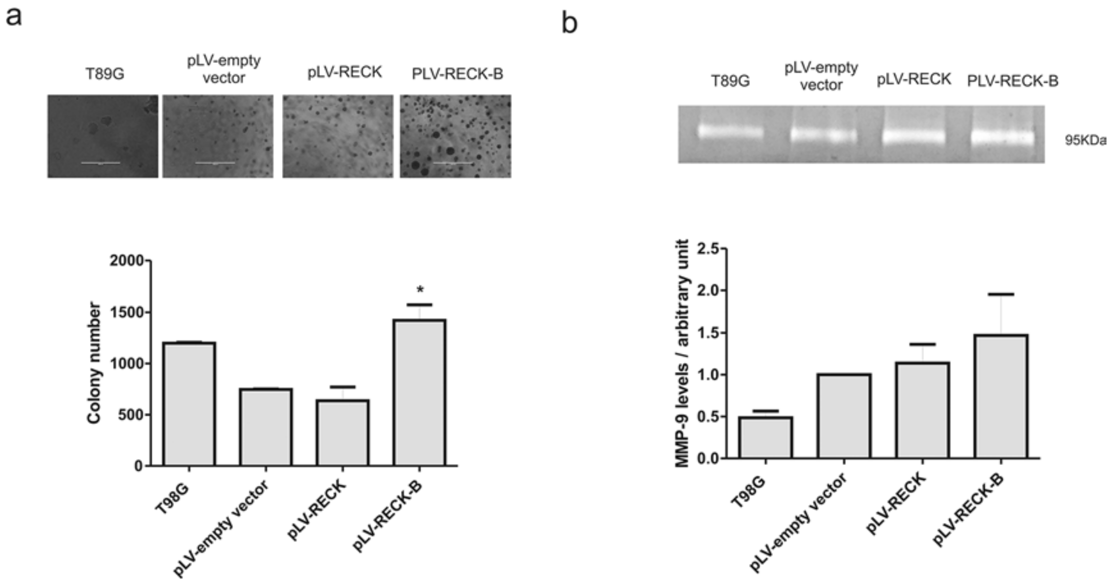

Supplementary Figure S2: Canonical RECK and RECK-B functional analysis in T98G cells. T98G cells overexpressing RECK and RECK-B, as well as the empty vector control, along with the parental cell line, were subjected to anchorage-independent clonal grow in semi-solid medium **a.** and gelatin zymography assay **b.** Results are represented as the mean with standard deviations from three independent experiments. ANOVA test followed by Tukey's test for *post hoc* comparison were used for statistical analysis. * represents $p < 0.05$.

Supplementary Table S1: Sequences of the primers used for detection and amplification of the *RECK* alternative isoforms

	Target Isoform	*Primer Sequences (5′ → 3′)*
Amplification	*RECK-B*	Forward -ATGGCGACCGTCCGGGCCTCTCTGCGAGGTGCGCTGCTCCTTCT<br>Reverse—TTTGGTCAGTTTGGTCAATCA
	RECK-I	Forward -ATGGCGACCGTCCGGGCCTCTCTGCGAGGTGCGCTGCTCCTTCT<br>Reverse—CTGCCATCTCCTTAGCTCCA
Real Time PCR	*RECK*	Forward—GTGCCGTGATGTATGTGAACAGATT<br>Reverse—GCAACAGATGTTTTAGTCGGGATTC
	RECK-B	Forward—TATTGCGCCTCTATTAGTCCACAA<br>Reverse—CAGCTTCATCAGGAGGCCTATC
	RECK I	Forward—ATGAGGAACCCAACGGATAGCAG<br>Reverse—GCCATCTCCTTAGCTCCAGCAGTA
5′ RACE PCR	*all*	GCAATAGCCAGTTCACAG
	all	CAGCGCACCTCGCAGAGA
3′RACE PCR	*RECK-B*	ACCCAACGGATAGGCCTCCT
	RECK-I	GGAACCCAACGGATAGCAGAT
